# The Talin Head Domain Reinforces Integrin-Mediated Adhesion by Promoting Adhesion Complex Stability and Clustering

**DOI:** 10.1371/journal.pgen.1004756

**Published:** 2014-11-13

**Authors:** Stephanie J. Ellis, Emily Lostchuck, Benjamin T. Goult, Mohamed Bouaouina, Michael J. Fairchild, Pablo López-Ceballos, David A. Calderwood, Guy Tanentzapf

**Affiliations:** 1 Department of Cellular and Physiological Sciences, University of British Columbia, Vancouver, Canada; 2 School of Biosciences, University of Kent, Canterbury, Kent, United Kingdom; 3 Department of Pharmacology, Yale University, New Haven, Connecticut, United States of America; 4 Carnegie Mellon University Qatar, Education City, Doha, Qatar; The University of Pennsylvania, United States of America

## Abstract

Talin serves an essential function during integrin-mediated adhesion in linking integrins to actin via the intracellular adhesion complex. In addition, the N-terminal head domain of talin regulates the affinity of integrins for their ECM-ligands, a process known as inside-out activation. We previously showed that in *Drosophila*, mutating the integrin binding site in the talin head domain resulted in weakened adhesion to the ECM. Intriguingly, subsequent studies showed that canonical inside-out activation of integrin might not take place in flies. Consistent with this, a mutation in talin that specifically blocks its ability to activate mammalian integrins does not significantly impinge on talin function during fly development. Here, we describe results suggesting that the talin head domain reinforces and stabilizes the integrin adhesion complex by promoting integrin clustering distinct from its ability to support inside-out activation. Specifically, we show that an allele of talin containing a mutation that disrupts intramolecular interactions within the talin head attenuates the assembly and reinforcement of the integrin adhesion complex. Importantly, we provide evidence that this mutation blocks integrin clustering in vivo. We propose that the talin head domain is essential for regulating integrin avidity in *Drosophila* and that this is crucial for integrin-mediated adhesion during animal development.

## Author Summary

Cells are the building blocks of our bodies. How do cells rearrange to form three-dimensional body plans and maintain specific tissue structures? Specialized adhesion molecules on the cell surface mediate attachment between cells and their surrounding environment to hold tissues together. Our work uses the developing fruit fly embryo to demonstrate how such connections are regulated during tissue growth. Since the genes and molecules involved in this process are highly similar between flies and humans, we can also apply our findings to our understanding of how human tissues form and are maintained. We observe that, in late developing muscles, clusters of cell adhesion molecules concentrate together to create stronger attachments between muscle cells and tendon cells. This strengthening mechanism allows the fruit fly to accommodate increasing amounts of force imposed by larger, more active muscles. We identify specific genetic mutations that disrupt these strengthening mechanisms and lead to severe developmental defects during fly development. Our results illustrate how subtle fine-tuning of the connections between cells and their surrounding environment is important to form and maintain normal tissue structure across the animal kingdom.

## Introduction

The formation and maintenance of three-dimensional tissue architecture requires fine-tuning of adhesion between cells and the extracellular matrix (ECM). Integrins are the principal family of cell-ECM adhesion receptors in metazoans and are comprised of an alpha and beta subunit [1]. The extracellular domain of integrins binds to the ECM and their cytoplasmic tail domains mediate linkage to the actin cytoskeleton via adapter proteins. The strength and stability of cell-ECM attachment varies in response to the cellular context: stable, long-lasting adhesion is used to preserve tissue architecture while short-term matrix attachment is used for dynamic processes such as cell migration during embryonic morphogenesis [2]. Thus, determining the strength and duration of adhesion to the ECM has important consequences for animal development and tissue maintenance.

The strength and duration of integrin binding to the ECM is controlled by two different mechanisms: by changing the conformation of integrins, and by regulating their clustering. Changes to the conformation of integrins, a process known as integrin activation, modulates the affinity of integrins for their ECM ligands. During activation, the heterodimer switches from a bent low-affinity state to an extended high-affinity state. In comparison, clustering of integrin receptors increases the avidity or accumulated strength of multiple integrin interactions with ECM ligands. Essential roles for both integrin activation and integrin clustering have been demonstrated in various systems and cell types. It is not known whether all cells where integrins are known to function use these two regulatory mechanisms. There are examples of cells and tissues that use regulation by: activation (for example platelets; [3], [4]), clustering (such as skin; [5]–[7]) or both (for example, several types of leukocytes; [4], [8]). An intriguing possibility is that there are tissue-specific contexts that require a particular mode of regulating integrin, either clustering or activation. However, the identification of such tissue-specific contexts requires the ability to abrogate clustering and/or activation, in vivo, across tissues and compare the observed phenotypes.

The large cytoplasmic protein talin is a central mediator of both integrin clustering and activation making it a perfect target for studies aiming to understand both processes. In particular, the N-terminal region of talin, known as the head domain, has been implicated in binding to and activating integrins, a process known as “inside-out” activation [9]. The talin head is composed of an atypical FERM (Band 4.1/Ezrin/Radixin/Moesin) domain that is made up of four lobular subdomains (F0, F1, F2, and F3;[10]). The F3 subdomain mediates direct interactions with the β−integrin cytoplasmic tail and is required to induce integrin activation [11], [12] but the other subdomains also contribute to integrin activation [9], [13]. The mechanisms by which the talin head domain mediates integrin activation have been studied extensively [14]. The model emerging from these studies is that plasma membrane interactions mediated via the F1, F2 and F3 subdomains, together with F3-dependent β-tail interactions, induce a change in angle of the β-integrin transmembrane domain relative to the membrane [15]–[18]. It is this movement that promotes the conformational changes that drive integrin activation. In comparison, although talin has an established role in inducing integrin clustering [19], [20] the mechanism that mediates this function has yet to be elucidated. Nonetheless, Saltel and co-workers [21] suggest based on their studies that clustering involves similar conformational changes in integrin to those that take place during activation.

Studies in *Drosophila melanogaster* have generated useful insight into the regulation of integrin function in vivo [1], [22]–[26]. This is because flies are particularly amenable to transgenic and mutational structure/function analysis. Also, there is an array of developmental processes during fly development that are integrin-dependent allowing for integrin function to be analyzed in diverse contexts. Previous studies in the fly have addressed the role of talin head mediated integrin activation in vivo. When introduced into fly talin, a mutation that abolishes the ability of the talin head to bind integrin via Integrin Binding Site 1 (IBS-1) in the F2-F3 domain (R367A in fly talin), resulted in a phenotype that was mild, consistent with slight weakening of the attachment between integrins and the ECM [25]. Furthermore, phenotypes were only in the muscle at prominent, well-characterized sites of integrin-mediated adhesion known as myotendinous junctions (MTJs). MTJs are sites where integrins mediate stable attachment between muscles and the overlying epidermis. Subsequent studies showed that a second, more C-terminal, integrin binding site (IBS-2) in talin is the main linker between integrins and the Intracellular Adhesion Complex (IAC) and that this interaction is of particular importance during morphogenetic events that require more dynamic adhesion [24]. Thus, it was posited that that the head domain is predominantly required for stabilizing rather than establishing Cell-ECM adhesion. These studies leave unresolved questions with respect to the role of talin-head dependent integrin activation, as the IBS-1 mutation used did not specifically abrogate activation, but rather completely disrupted binding between talin head and integrin. Intriguingly, work using insect cell-culture argues that the canonical talin-head induced integrin activation does not occur in *Drosophila* [27]. Overall, the existing body of data suggests the talin head contributes in novel and as of yet undetermined ways to regulating integrin-mediated adhesion in *Drosophila*.

Here, we utilize a structure/function approach to investigate the role of the talin head domain in the context of the developing fly embryo. We use targeted mutations in talin that abolish talin head-mediated integrin activation while leaving all other functions of the talin head intact. We are thus able to confirm that canonical talin head-mediated inside-out activation is indeed not essential for fly development. Importantly, we identify a point mutation in the talin head that phenocopies complete deletion of the talin head, and interferes with the reinforcement of cell-ECM adhesion. A key feature of this mutation is that it not only disrupts talin head domain-mediated integrin activation but also impinges on integrin clustering. Biochemical analysis of provides a mechanistic basis for the phenotype underlying the mutation, identifying an essential intramolecular interaction between the F2 and F3 subdomains of talin. Our results suggest that a major function of the talin head is to induce integrin receptor clustering, and to promote adhesion maturation. Moreover, we provide genetic evidence demonstrating that clustering is the primary mechanism by which integrin function is regulated in developing fly embryos.

## Results

### Integrin activation is not essential for fly embryogenesis

**Figure 1:**
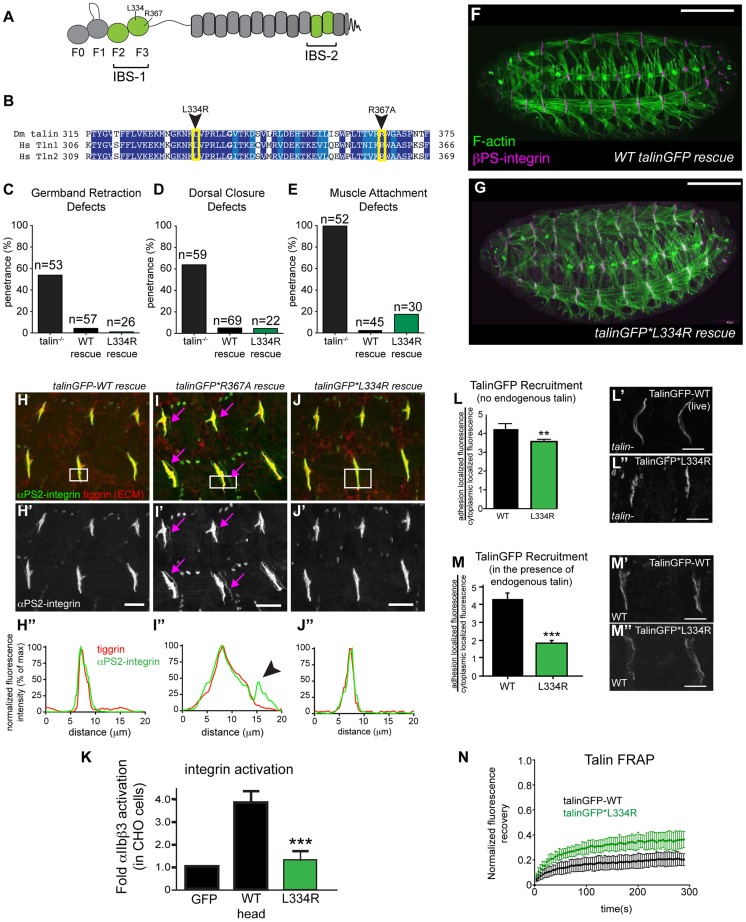
Integrin-binding to the talin head, but not integrin activation, is required for muscle attachment. (**a**) Schematic of key domains in talin for this study. The talin head is contains an N-terminal atypical FERM domain [10] and a C-terminal rod domain comprised of 13 helical bundles [59]. (**b**) Alignment of residues 325–375 of fly talin F3 domain with human talin isoforms. Dark blue indicates identical residues between homologues, lighter blue indicates similar residues. The mutations utilized to study integrin activation are indicated with an arrowhead. (**c–e**) Integrin-dependent phenotypes germband retraction (c), dorsal closure (d) and muscle attachment (e) were assayed in talin-null embryos, WT talinGFP-rescued embryos, and talinGFP*L334R-rescued embryos. Apart from mild muscle detachment in about 20% of embryos, the talinGFP*L334R transgene was able to rescue all phenotypes such that the embryos hatched to the larval stages. (**f–g**) Maternal zygotic talin null embryos rescued with either full-length WT talinGFP transgene (f) or talinGFP*L334R mutant transgene (g) and stained for F-actin (green) and βPS-integrin (magenta). (**h–j**) MTJs of talin null embryos rescued with either talinGFP-WT (**h**), talinGFP*R367A (**i**), talinGFP*L334R (**j**). Embryos were stained for anti- **α**PS2-integrin (green in **h–j**; grey in **h′–j′**) and tiggrin, a Drosophila ECM molecule (red in **h–j**). (**h″–j″**) Average intensity profiles for integrin and tiggrin across the widths of the boxed areas in h–j. Tiggrin and integrin completely overlapped at MTJs in WT talin rescue embryos (h″), but were separated from one another in talin-null embryos rescued with talinGFP*R367A (i–i″). Overlap between tiggrin and integrin was maintained in talin-null embryos rescued with talinGFP*L334R (j–j″). The pink arrowheads mark the sites of separated integrin and ECM signal. (**k**) Activation of human integrins by fly talin head constructs was measured in CHO cells. The L334R mutation was sufficient to abrogate integrin activation. (**l–m**) Recruitment of ubi-promoter driven full-length WT talinGFP and talinGFP*L334R to sites of adhesion was assayed in talin null (l) and in wild-type embryos (m). Compared to WT TalinGFP, TalinGFP*L334R was well recruited in a background devoid of any endogenous talin (l–l″; **p<0.01), but competed less well in the presence of endogenous talin and was only weakly recruited to sites of adhesion compared to WT, which was robustly recruited (m–m″; ***p<0.001). (**n**) FRAP experiments on WT talinGFP and talinGFP*L334R reveal that talinGFP*L334R is much less stable at sites of adhesion than WT talinGFP. Scale bars: f–g  = 100 µm; h–j;l–m = 20 µm.

We sought to introduce a mutation into the talin head that disrupted its ability to activate integrins but not other aspects of its function. We relied on the extensive knowledge of talin structure generated by previous NMR and crystallographic analysis of the talin-head interaction with integrin in order to do this. Previous studies identified a mutation (Fig. 1a–b; L325R in talin1, L331R in talin2) in mammalian talin that specifically abrogates the integrin-activating function of talin, but does not substantially affect the ability of the talin head to bind to integrin [28]. When this mutation is introduced into the talin head, it blocks the conformational change in integrin that drives activation. The residue identified specifically attenuates the interaction between the talin head and integrin at the membrane proximal region of the β-integrin cytoplasmic tail, while maintaining the interaction between the talin head and the distal regions of the β-integrin cytoplasmic tail [28]. We introduced this mutation into fly talin (L334R) to study its effects.

Our lab has previously developed and extensively utilized a protocol to assess the effect of point mutations in fly talin. This protocol relies on the dominant-female sterile germline clone technique [29];(see Materials and Methods) to remove all the endogenous talin from fly embryos. To replace the endogenous talin we used ubiquitously expressed full-length talin rescue constructs, either wild-type (WT) talinGFP, or talin point mutants. The WT talinGFP construct rescued the embryonic lethality associated with loss of talin. Surprisingly, we found that talinGFP*L334R-rescued embryos were in some cases indistinguishable from WT talinGFP-rescued embryos; we observed many L334R-rescued embryos hatching to the larval stages (Fig. 1c–g). Talin-dependent morphogenetic movements germband retraction (GBR) and dorsal closure (DC) were not affected by the L334R mutation (Fig. 1c,d,g). However,, we observed that approximately 20% of late stage 17 embryos possessed a form of muscle detachment defect (Fig. 1e; Supplemental Fig. S1). Previous work on talin*R367A, a mutation that abrogates talin binding to integrin via its IBS-1 domain and consequently blocks activation, also revealed late muscle detachment defects (Supplemental Fig. S1; [24], [25]). However, the late muscle defects in talin*R367A mutant were stronger and more penetrant than those of the talinGFP*L334R-rescued embryos (Supplemental Fig. S1). A hallmark of the talin*R367A phenotype is detachment of integrins from the ECM, marked by staining for the protein Tiggrin (Fig. 1i; [24], [25]). In contrast, in talinGFP*L334R-rescued embryos, we could not detect detachment of integrins from the ECM (Fig. 1j). Similar to WT talinGFP-rescued embryos (Fig. 1h), they exhibited complete overlap of integrin and ECM signal at MTJs. A possible explanation for the difference between the R367A and L334R mutation would be that the L334R mutation might not block integrin activation in flies. To directly test this possibility, we used a cell culture based activation assay to confirm that the L334R mutation indeed blocked the ability of the talin head to activate integrins. GFP-tagged fly talin head constructs, either WT or L334R, were transiently expressed in CHO cells that stably express human αIIbβ3 integrins. Activation was assessed using an established flow cytometry-based assay to quantify activation of αIIbβ3 integrins. As was previously shown [27], fly talin robustly activates human integrins when expressed in cell culture (Fig. 1k). In comparison, we found that the L334R mutation in the talin head abrogated integrin activation (Fig. 1k).

Consistent with the mild phenotype observed in talinGFP*L334R-rescues, we found a number of subtle differences in sub-cellular localization and dynamics between WT and L334R mutant talin. First, recruitment of talinGFP*L334R to sites of integrin-mediated adhesion was slightly reduced compared to WT talinGFP (Fig. 1l). This effect was more pronounced in the presence of endogenous talin suggesting that the mutant protein is outcompeted by the untagged WT protein for integrin binding (Fig. 1m). Second, we found that the turnover of talinGFP*L334R was elevated compared to that of WT talinGFP (Fig. 1n), when we employed a Fluorescence Recovery After Photobleaching (FRAP) protocol that we developed to analyze the stability of components of the integrin adhesion complex in sites of Cell-ECM attachment in the fly muscle [30], [31]. Taken together, these data suggest that talin head-mediated integrin-activation, or at least L334-dependent activation, is dispensable for most of fly embryogenesis but does play a small role late in development in stabilizing adhesion to maintain tissue architecture.

### The talin head is required for integrin-dependent morphogenesis and muscle attachment

**Figure 2:**
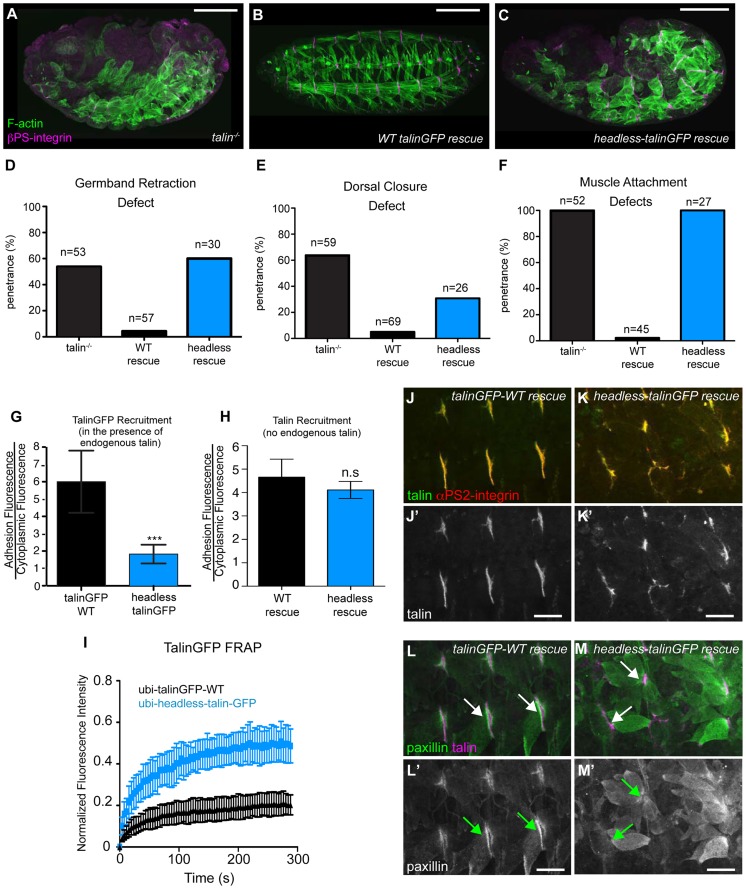
The talin head is essential for integrin function in *Drosophila*. (**a–c**) Maternal-zygotic talin null embryos (shown in a) rescued with either full-length WT talinGFP transgene (b) or headless talinGFP transgene (c) and stained for F-actin (green) and integrin (magenta). (**d–f**) Integrin-dependent phenotypes germband retraction (d), dorsal closure (e) and muscle attachment (f) were assayed in talin-null embryos, WT-talin-rescued embryos, and headless-talin-rescued embryos. The talin head was required for all three processess assayed. Scale bar = 100 µm. (**g–h**) Recruitment of ubi-promoter driven, GFP-tagged full-length WT talin and headless talin to sites of adhesion was assayed in wild-type embryos (g) and in a talin null background (h). In a WT background, headless-talinGFP competed less well with endogenous talin and was only weakly recruited to sites of adhesion compared to WT (***p<0.001); in the absence of endogenous talin, headless-talin was well recruited to sites of adhesion. (**i**) FRAP experiments on talinGFP-WT and headless-talinGFP reveal that headless talin is much less stable at sites of adhesion than talinGFP-WT. (**j–m**) Confocal z-stacks of stage 17 maternal/zygotic-mutant embryos rescued with either full length WT talin (j,l) or headless-talin (k,m). (**j–k**) adhesions stained for talin (green in j–k; grey in j′–k′) and integrin (red in j–k). In the absence of the talin head, talin was still well recruited. (**l–m**) Muscles stained for talin (magenta in l–m) and paxillin (green in l–m; grey in l′–m′). Paxillin was not well recruited to adhesions. Scale bars: a–e = 100 µm; j–m = 20 µm.

We sought to identify roles of the talin head beyond integrin binding and activation. To this end, using the same approach described above, we replaced endogenous talin with a ubiquitously expressed construct that deletes the talin head (residues 1–448) but leaves the rest of talin intact: headless-talinGFP. Importantly,head deletion resulted in severe phenotypes resembling complete loss of talin (Fig. 2a–c). GBR and DC were severely disrupted (Fig. 2d–e), as was stable muscle attachment to the ECM (Fig. 2f). While the headless-talinGFP localized poorly in the presence of endogenous talin it exhibited robust localization in talin null embryos (Fig. 2g, h) even though its overall expression appeared somewhat lower compared to wild type (Supplemental Fig. S2). Therefore headless-talinGFP was able to retain functional interactions that supported recruitment to sites of adhesion. Nonetheless, FRAP analysis showed that headless-talinGFP was substantially less stable at sites of adhesion (Fig. 2i). In addition, the adhesion complex that is normally recruited to sites of adhesion by talin ([25], [32]; Fig. 2j,l), was absent or severely reduced in headless-talinGFP rescue embryos (Fig. 2k,m). These results show that deletion of the head results in severe defects in recruitment and stabilization of the adhesion complex; consequently, loss of talin head function blocks talin-dependent morphogenetic events.

### *rhea^17^* encodes a missense mutation in the talin head, G340E, required for talin function

**Figure 3:**
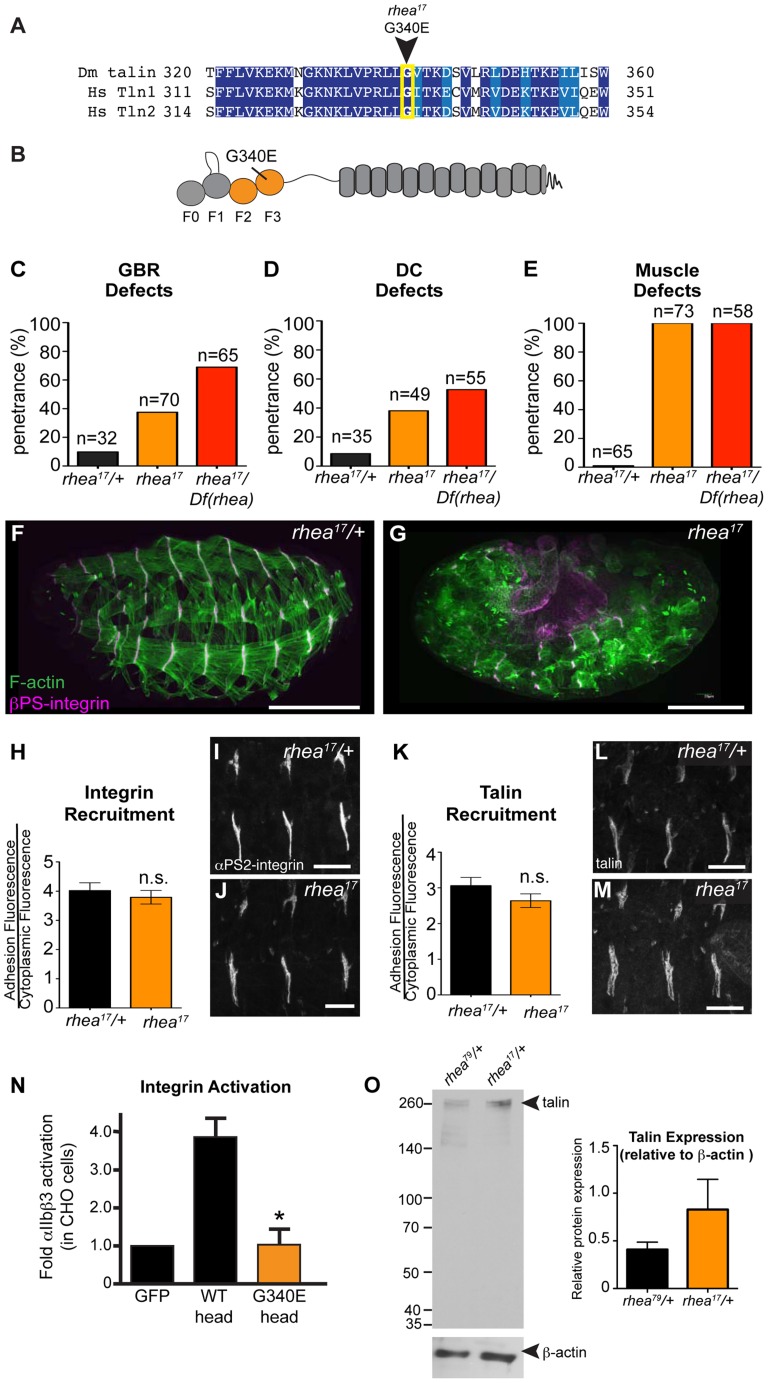
*rhea^17^* encodes a hypomorphic talin protein which disrupts talin head function. (**a–b**) The *rhea^17^* allele is characterized by a missense mutation in a conserved glycine residue in the F3 lobe of the talin head FERM domain, G340E. (**c–g**) Whole mount stage 17 embryos stained for F-actin (green) and integrin (magenta) reveal that *rhea^17^* mutant embryos (g) harbour severe morphogenetic phenotypes in GBR (c) and DC (d), as well as muscle detachment defects (e) compared to WT heterozygous embryos (f). Phenotypic analysis of rhea17 over the rhea79 deficiency increased the penetrance of all phenotypes. (**h–j**) αPS2-integrin recruitment was measured in WT (h,i) and *rhea^17^* (h,j) stage 16 embryonic muscles stained for integrin. Integrin was recruited at WT levels in *rhea^17^* embryos. (**k–m**) Talin recruitment was measured in WT (k,i) and *rhea^17^* (k,m) stage 16 embryonic muscles stained for talin. Talin was well recruited in *rhea^17^* embryos. (**n**) Activation of human integrins by fly talin head constructs was measured in CHO cells. The G340E mutation was sufficient to abrogate integrin activation compared to WT. (**o**) Quantitative Western blot analyses of relative levels of talin normalized to beta-actin levels in flies heterozygous for either the *rhea^79^* talin null mutation (left lane) or the *rhea^17^* mutant allele (right lane). Scale bars: f–g = 100 µm; i–j; l-m = 20 µm.

Thus far, we have shown that talin-head mediated integrin activation plays only a minor role in talin function during fly develeopment and that despite this, the head domain has other essential functions in mediating integrin-based Cell-ECM adhesion. To uncover the mechanism underlying additional roles for talin we turned to a previously isolated allele of talin, *rhea^17^*. This allele encodes a talin protein containing a missense mutation in the talin head and importantly, produces a phenotype that is similar to that observed when the talin head is deleted in full (Fig. 2b, Fig. 3f). The *rhea^17^* allele was originally uncovered in a screen for dominant enhancers of a hypomorphic integrin allele [32]. We sequenced the *rhea^17^* allele (see materials and methods and Supplemental Fig. S3) and found that it contains a mutation that replaces a conserved glycine, G340 (G331 in mammalian talin1, G334 in mammalian talin2), to a glutamate (G340E; Fig. 3a–b). Phenotypic analysis of embryos homozygous for this mutation (Fig. 3f–g) showed that integrin-dependent morphogenetic processes GBR and DC as well as stable muscle attachment to the ECM were severely disrupted compared to heterozygous controls (Fig. 3f–g, c–e). Interestingly, embryos that had a GBR phenotype were more likely to have a DC phenotype. For example, while 38% of the total population of *rhea^17^* mutant embryos analyzed displayed DC defects, amongst those that also have GBR defects, the proportion of embryos with DC defects increased to about 63%. This connection seems likely, because the morphology of the amnioserosa, an extra-embryonic tissue required for both GBR and DC [33], [34], is defective as a result of GBR failure. To ensure that the phenotypes we observed in embryos homozygous for the *rhea^17^* mutation were not due to the accumulation of background mutations, we analyzed the phenotype of embryos trans-heterozygous for the *rhea^17^* allele and a talin null allele (*rhea^17^/Df*). This revealed an even stronger phenotype, suggesting that the phenotype observed in the *rhea^17^* homozygous mutants is not due to background mutations. Furthermore, this implied that the *rhea^17^* allele is a hypomorphic mutation that retains some functionality in comparison to complete loss of talin (Fig. 3c–e). Of note, the GBR phenotype of *rhea^17^*/*Df* embryos was stronger than that of *rhea^79^* talin null mutant embryos (Fig. 3c). It is thus possible that the *rhea^17^* might be acting in a dominant negative fashion in this process. This is consistent with what we have previously observed for some mutations in integrin that give rise to stronger GBR phenotypes than the loss of function mutants [35]. Another possibility is that the *rhea^79^* allele may have accumulated background mutations that slightly suppress the talin null phenotype.

A possible explanation for the strong phenotype observed in *rhea^17^* mutant embryos was that the mutation compromises the stability of talin protein such that functional defects observed could arise from insufficient levels of talin protein. However, analysis of *rhea^17^* embryos revealed that the G340E mutation did not affect either the localization to or levels of, both integrin (Fig. 3h–j) and full-length talin at sites of adhesion at MTJs (Fig. 3k–m). Furthermore, Western blot analysis did not reveal any detectable degradation products associated with the *rhea^17^* mutation (Fig. 3o). Finally, side-by-side analysis of embryos heterozygous for either the *rhea^17^* allele or the *rhea^79^* talin null allele demonstrated that there was about twice as much talin protein in the *rhea^17^/+* embryos compared to *rhea^79^/+*. This result indicated that full-length talin protein product from the *rhea^17^* allele was expressed at levels comparable to the wild type allele, demonstrating that the talin protein containing the G340E mutation is stable and sufficiently expressed (Fig. 3o).

### The G340E mutation abrogates integrin activation

Since the full-length G340E talin encoded by the *rhea^17^* allele is able to localize to sites of adhesion we asked whether this mutation blocked the ability of the talin head to bind to and activate integrins. To this end, we again employed a flow cytometry-based αIIbβ3 integrin activation assay. This showed that that the G340E point mutation, much like the L334R mutation, blocked the ability of the talin head to activate integrins (Fig. 3n).

### The G340E mutation affects integrin clustering

**Figure 4:**
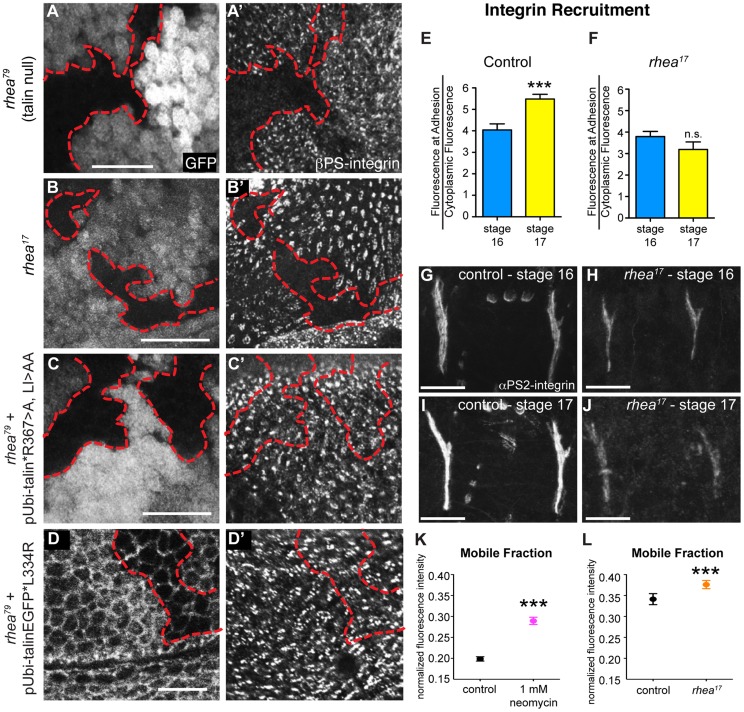
*rhea^17^* disrupts integrin clustering. (**a–a′**) Clones of cells lacking talin, marked by the absence of GFP (a), failed to cluster integrins into adhesions (**a′**). (**b,b′**) Clones of cells expressing the *rhea^17^* mutant allele of talin (marked by absence of GFP in **b**) also failed to cluster integrins (**b′**). (**c–d**) Expression of a full length talin point mutant that specifically disrupts IBS-1 binding (c, talinGFP*R367A, LI>AA, see Ellis et al, 2011) or that specifically disrupts integrin activation (talinGFP*L334R) restored integrin adhesions (**c′, d′**) within the clones of cells (arrow) lacking endogenous talin and the GFP marker (**c,d**). The red outline demarcates the position of the clones. Note that in **d**, cell outlines are also marked with GFP due to localization of the talinGFP*L334R protein to basolateral membranes. (**e–j**) Recruitment of integrins to MTJs was measured in stage 16 and stage 17 for both control (e,g,i) and *rhea^17^* mutant embryos (f,h,j). In contrast to control embryos (***p<0.001), *rhea^17^* mutant embryos did not exhibit an increase in integrin recruitment to MTJs during this developmental transition. (**k–l**) FRAP analysis revealed the mobile fraction of integrin-YFP was higher than respective controls in embryos treated with neomycin (**k**; ***p<0.001) or in *rhea^17^* zygotic mutant embryos (**l**; ***p<0.001). Since these two FRAP experiments employed different genetic backgrounds and protocols in preparation for FRAP (ie. embryos in **k** were subjected to a drug delivery protocol), they necessitated two separate controls. In **k**, the control was established from vehicle-treated wild type embryos expressing integrin-YFP. In **l**, the controls were taken from heterozygous talin mutant embryos. Scale bars: a–d = 10 µm; g–i; h–j = 20 µm.

The phenotype observed in *rhea^17^* embryos cannot be explained by a defect in integrin activation alone since our data demonstrates that blocking activation by itself does not give rise to a severe phenotype (Fig. 1). Therefore, we hypothesized that the underlying cause of the *rhea^17^* phenotype is due to a defect in another function associated with talin: integrin clustering [19], [21]. Integrin clustering in the fly can be assessed using a well-established *in vivo* assay in the context of the fly imaginal wing disc epithelium [25], [32], [36]. In the imaginal wing disc integrins mediate adhesion between the epithelial layers and form discrete puncta that colocalize with other adhesion complex components including talin, on the basal surface of the epithelium [32]. In the absence of talin these clusters fail to form, indicating a role for talin in integrin clustering (Fig. 4a; [32]). Interestingly, clonal patches of homozygous *rhea^17^* mutant cells also failed to form integrin clusters (Fig. 4b). In comparison, neither the R367A mutation, nor the L334R mutation, disrupted integrin clustering (Fig. 4c–d; [25]). These results are in line with the hypothesis that the G340E mutation in *rhea^17^* directly impinges on the ability of talin to cluster integrins.

If the G340E mutation impacts integrin clustering, we predicted that this would affect the recruitment of integrins to sites of adhesion. To test this idea we analyzed integrin recruitment to MTJs. Consistent with previous reports [37], integrin recruitment to MTJs exhibited a substantial increase between embryonic stages 16 and 17 in control WT embryos (Fig. 4e,g,i), but in *rhea^17^* embryos, this increase did not occur (Fig. 4f,,h,j). While this result hints at a defect in integrin clustering, it only provides indirect support for this hypothesis. Furthermore, to more directly test the role of clustering we utilized neomycin, a reported inhibitor of integrin clustering that works by sequestration of PI(4,5)P2 membrane phospholipids [19], [38], [39]. It was previously shown that neomycin treatment results in increased turnover of integrins at focal adhesions [19]. FRAP analysis of MTJs revealed that the mobile fraction of integrin-YFP increased by over 45% at sites of adhesion in embryos treated with neomycin compared to control vehicle-treated embryos (Fig. 4k). The mobile fraction of integrin-YFP also increased over heterozygous controls in *rhea^17^* mutant embryos (Fig. 4l). Overall, we present three lines of evidence which taken together, support the idea that clustering is disrupted by the presence of the G340E mutation in talin.

### *rhea^17^* mutant embryos display defects in adhesion complex reinforcement

**Figure 5:**
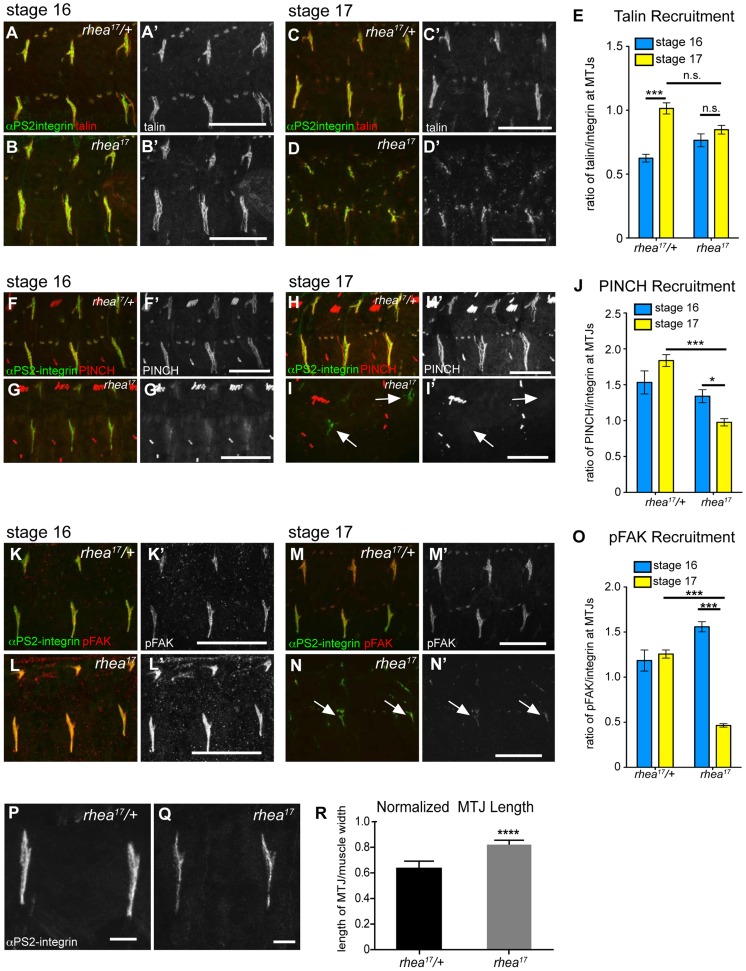
*rhea^17^* disrupts adhesion complex reinforcement and adhesion consolidation. WT and *rhea^17^* embryonic muscles stained for talin (red in a–d; grey in a′–d′) and integrin (green in a–d) at stage 16 (a–b) and stage 17 (c–d). (e) The recruitment of talin to adhesions (normalized to integrin levels; see materials and methods) was comparable between WT and *rhea^17^* in stage 16 embryos. However, although talin was maintained at sites of adhesion, its recruitment was not reinforced in *rhea^17^* embryos in stage 17 embryos (e). (**f–j**). WT and *rhea^17^* embryos stained for integrin (green in f–i) and PINCH (red in f–i, grey in f′–i′) at stage 16 (f–g) and stage 17 (h–i). PINCH recruitment was not reinforced in stage 17 *rhea^17^* embryos as determined by measuring the ratio of anti-PINCH fluorescence intensity relative to integrin intensity at MTJs. (j; see Materials and Methods). (**k–o**) WT and *rhea^17^* embryos stained for integrin (green in k–n) and pFAK (red in k–n; grey in k′–n′). pFAK recruitment was not reinforced in stage 17 *rhea^17^* embryos as determined by measuring the ratio of anti-pFAK fluorescence intensity relative to integrin intensity at MTJs (o; see Materials and Methods). (**p–r**) MTJ length was measured in control heterozygous (p) and *rhea^17^* mutant (q) embryos (see materials and methods). MTJs were significantly longer in *rhea^17^* mutants compared to control embryos (****p<0.0001). Scale bars: a–n = 50 µm; p–q = 10 µm.

Both the fact that the talin head was required for adhesion complex assembly (Fig. 2), and that the G340E mutation interfered with integrin clustering (Fig. 4), led us to hypothesize that the *rhea^17^* mutation might give rise to defective adhesion complex assembly and maintenance. Since MTJs grow and mature over several hours of embryonic development (stages 16–17), they serve as a useful system to study the maturation of integrin-mediated adhesions. In WT embryos talin is localized at MTJs as they form during stage 15 and then undergoes substantial enrichment between stages 16 and 17 as Cell-ECM adhesions are consolidated and reinforced (Fig. 5a,c,e). Recruitment of other adhesion complex components including PINCH (Fig. 5f,h,j) and pFAK (Fig. 5k,m,o) also occurred at stage 16 and was maintained through stage 17. In *rhea^17^* embryos, although talin is well recruited to MTJs by stage 16, its recruitment is not reinforced in stage 17 (Fig. 5b,d,e). Strikingly, other adhesion complex components such as PINCH (Fig. 5g,i,j) and pFAK (Fig. 5l,n,o) were also initially recruited to MTJs at stage 16 at levels comparable to WT, but by stage 17, the levels were drastically reduced. Intriguingly, we found that MTJs are longer in *rhea^17^* embryos compared to WT controls, further suggesting a failure in adhesion maturation and consolidation (Fig. 5p–r). Altogether, these data demonstrate that the G340E mutation in the talin head impinges on the ability to reinforce integrin-mediated adhesions, consistent with a defect in adhesion maturation, leading to the breakdown of the Cell-ECM adhesion.

### The G340E mutation disrupts the interface between subdomains F2 and F3 in the talin head

**Figure 6:**
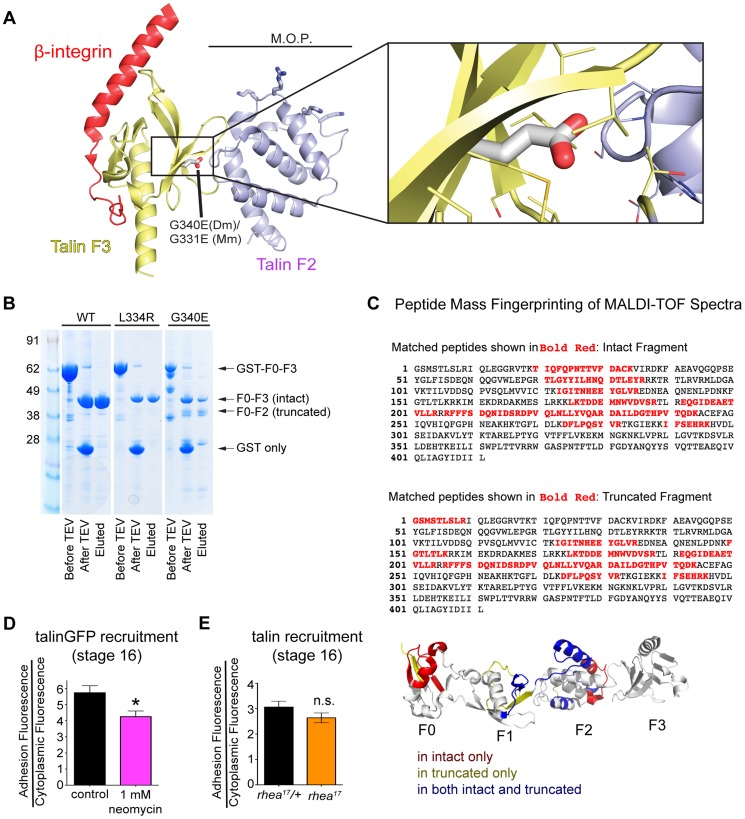
G340 maintains an intermolecular interaction between F2 and F3 that couples their activity. (**a**) The conserved role of G340 (G331 in mammalian talin2, shown here) is to stabilize the domain orientation of F2 and F3 (a), which work together to induce integrin activation and stabilize that talin head at the plasma membrane. Modelling based on known structures (see [15]) of mouse talin2 and the integrin cytoplasmic tail suggests that the G340E mutation would disrupt the tight apposition of F2 and F3, thus allowing them to behave as independent modules. (**b**) In vitro expression of WT (left lane), L334R (middle lane), and G340E (right lane) constructs reveals proteolytic sensitivity of G340E compared to WT and L334R. (**c**) MALDI-TOF mass spectrometry and peptide mass fingerprinting were used to identify that the sequence of the truncated fragment of the talin head observed in (b) corresponded to the F0-F2 domains indicating F3 was often cleaved in the G340E mutant. (**d–e**) The recruitment of talin was measured in neomycin treated embryos (d) and in *rhea^17^* embryos (e). Talin recruitment was significantly reduced in neomycin-treated compared to controls (*p<0.05). In contrast there was no such reduction in the *rhea^17^* embryos suggesting the G340E mutant talin protein interacts with the membrane as well as the WT protein.

We have shown that talin protein in *rhea^17^* mutants fails to cluster integrins and reinforce integrin-mediated adhesions. However, the data presented so far does not explain the mechanism by which this effect is mediated. When put into the context of the large body of knowledge that exists about the structure of the talin head, the nature of molecular lesion in *rhea^17^* provides some intriguing hints about this mechanism. Specifically, the G340E mutation is expected to disrupt the coordinated movement of the F2 and F3 domains which is essential for activation and clustering. Structural modeling of the talin head, using the solved crystal structure of the mouse talin head in complex with β1-integrin ([15]; PDB number 3G9W), predicted that residue equivalent to G340 (G331) is located on the surface of F3 at its interface with F2 (Fig. 6a, inset). In the WT talin head this glycine allows the close packing of these two sub-domains. However, substitution for a glutamate inserts a charged carboxyl group into this close gap disrupting the fixed orientation of the F2 and F3 domains, which should allow them to move independently of one another. Since it has been proposed that a tri-partite interaction between integrin, talin head, and the phospholipid bilayer is required to facilitate stable adhesion and to promote inside-out signaling, it is quite possible that disrupting the coordination of F2 and F3 would destabilize these interactions. Consistent with such an effect the G340E mutation rendered the talin head domain proteolytically sensitive to cleavage of the F3 subdomain from the F0-F2 subdomains (Fig. 6b). Furthermore, we used MALDI-TOF mass spectrometry and peptide mass fingerprinting to confirm that the cleaved fragment we observed was indeed the F0-F2 domain, indicating that the F3 had been lost (Fig. 6c). This *in vitro* evidence is in line with the assertion that F2 and F3 become structurally uncoupled from one another when G340 is mutated.

Structural analysis of the mammalian talin head has shown that the orientation of the F2-F3 domains is fixed and that this has important implications for the talin function, for example during inside-out activation [15]. It was shown previously that a cluster of basically charged residues in the F2 domain, the membrane orientation patch (MOP; Fig. 6a), play a key role in integrin activation via electrostatic interactions with the plasma membrane. In order to achieve optimal interaction of the MOP with the phospholipid headgroups, the F2-F3 module needs to reorient relative to the membrane. Multiscale molecular dynamics simulations suggest that this reorientation results in a ∼20° change in the tilt of the β-integrin transmembrane domain [16] leading to integrin activation via dissociation of the α-integrin and β-integrin transmembrane domains. Therefore, if the effect of the G340E mutation on clustering is due to disruption in the process of tilting and dissociation of the α-integrin and β-integrin transmembrane domains than mutations that specifically disrupt this process should also impinge on clustering. We therefore tested three mutations that have been proposed to have such an effect: first the L334R mutation in talin which was suggested to block the change in tilt angle of the β-integrin transmembrane domain [28]. Second, we tested two mutations in integrin previously suggested to promote dissociation of the α-integrin and β-integrin transmembrane domains, D807R (D723R in β3 integrin; [35], [40], [41]) and G792N (G708N in β3 integrin;[35], [42]). We found that clustering was similar to wildtype for all three mutations when we replaced endogenous talin or integrin with the mutant proteins (Fig. 4d and Supplemental Fig. S4). We therefore did not find evidence that supports the idea that the G340E mutation is defective in clustering due to interference with tilting and dissociation of the α-integrin and β-integrin transmembrane domains.

Another possibility is that the G340E mutation disrupts the ability of the F2-F3 domains to bind the plasma membrane, which effectively reduces the affinity of the interaction between the talin head and integrin [15]. To test if specific membrane interactions might play a role in talin recruitment to sites of integrin-mediated adhesion, we again used the drug neomycin. Neomycin sequesters PIP2, which is the predominant phospholipid type that the talin head interacts with at the plasma membrane [15]. Consistent with this idea, we found that with neomycin treatment the recruitment of talinGFP to MTJs in embryos was significantly reduced compared to controls (Fig. 6d). In comparison, we failed to see a reduction in recruitment with the G340E mutation suggesting that the phenotype arises via a different mechanism than simple disruption of talin head's interaction with the plasma membrane (Fig. 3k–m; Fig. 6e). This result implies that F2-F3 coordination could be important for integrin clustering through a mechanism other than by ensuring membrane embedding of the talin head domain.

## Discussion

Here we define a function for the talin head domain in regulating integrin adhesion by modulating clustering and therefore avidity for the ECM in *Drosophila*. Our analyses support several notable conclusions: (1) canonical talin-dependent integrin activation is largely dispensable for integrin function in flies; (2) a major function of the talin head is to promote integrin clustering and adhesion maturation; (3) long-term maintenance of the integrin adhesion complex may depend on a novel, previously uncharacterized mechanism that coordinates the spatial arrangement of F2 and F3; (4) disruption of clustering and/or adhesion reinforcement leads to severe defects in integrin-mediated processes during fly embryogenesis.

It is established that modulating integrin affinity by the talin head is a key mechanism of integrin regulation in mammalian systems [3], [11], [12], [43]. However, previous work suggested that this mechanism was not a major player in the fly [20], [25], [27]. Our study provides a possible explanation reconciling these results by suggesting that the major regulatory function of the talin head in flies is in modulating avidity rather than affinity. Furthermore, we show that the regulation of integrin clustering underlies reinforcement of Cell-ECM adhesions to drive their maturation during a key developmental transition. Multiple studies in a variety of cell types have shown that the modulation of avidity is important for integrin function in different contexts [4], [7], [8], [44]–[46]. For example, in leukocytes, it has been shown that the formation of integrin microclusters precedes ligand binding [45]. Interestingly, also in leukocytes, activated integrins are unable to mediate stable adhesion to ligand if the lateral mobility of integrins is restricted implicating a key role for integrin clustering in stable adhesions [44]. Furthermore, in both platelets and leukocytes, defective regulation of integrin clustering rather than integrin affinity has been shown to underlie the severe phenotypes caused by loss of kindlin, thus exemplifying the critical role of integrin clustering [47]. We would therefore argue based on our data, and the work of others that avidity regulation can in some instances be the main method of regulating integrin function.

The work we present here fits very well with the conclusions of two cell-culture based studies from Wehrle-Haller and co-workers that explore the role of talin in regulating integrin avidity [19], [21]. First, Cluzel et al used quantitative live imaging approaches to demonstrate that the formation of integrin clusters required the talin head and integrin [19]. Similar to what we observed, adhesion maturation (defined by recruitment of actin and other integrin adhesion complex proteins) only occurred following talin-head dependent clustering of integrins [19]. Furthermore, consistent with our data, their results also indicated that integrin clustering leads to the stabilization of integrin-mediated adhesions [19].

Second, to elucidate the molecular mechanisms by which the talin head supports integrin clustering, Saltel et al used quantitative live imaging, mutational analysis, and computational structural modeling approaches [21]. Based on their findings, they propose that the ability of the talin head domain to promote clustering relies on two separate interactions: between the F2-F3 subdomains of the head and the plasma membrane, and between F3 and the β-integrin cytoplasmic tail [21]. As a result both the F2 and F3 subdomains were equally required for integrin clustering; mutations that disrupted either the F2-F3/membrane interface or the F3/integrin interaction abrogated not only clustering but also focal adhesion maturation [21]. Our results support these findings in a physiological context as our analysis suggests that coordination between the F2 and F3 domains of the talin head is important for integrin clustering and adhesion reinforcement. Furthermore, we identify a specific conserved residue that may mediate this function. In general, the striking similarity between our data, which is derived from very different systems than those used by Cluzel, Saltel and co-workers, supports the notion that the role of talin and the talin head domain in regulating clustering is a basic conserved mechanism that may be important in various cell types.

What is the possible effect of the G340E mutation we describe? We envision two possibilities. First it could be that it is required to coordinate conformational changes within the F2 and F3 domain. Such coordination is required to ensure that concomitant with F2 binding to the plasma membrane through its membrane orientation patch, the F3 is able to bind to the integrin cytoplasmic tail to induce tilting ([15], [16], [18], [48]). However, we were unable to obtain results that support a role for tilt in regulating clustering in the fly, although we cannot discount this possibility. Secondly, the G340E mutation is in the F3 domain of talin in an area of the protein required to bind to the plasma membrane in such way so as to maximize its affinity for integrin [16], [48]. Therefore it is plausible that the G340E mutation might affect the affinity of talin binding to the integrin by weakening the interaction between the talin head and the plasma membrane. Such reduced affinity would lessen the capacity of talin to experience and transduce the forces necessary for clustering and maturation, since increased force would disrupt the talin-integrin bond. However, our results suggest that the G340E mutation, and disrupted F2-F3 coordination, might have a different consequence than weakening of the membrane-integrin-talin interaction. Specifically, in *rhea^17^* embryos, we did not observe the reduction in talin recruitment to sites of adhesion that might be expected if the G340E mutation affects talin binding to the membrane. Therefore, elucidating how proper F2-F3 coordination might contribute to integrin clustering remains an intriguing question for future study.

An intriguing difference between our results and previous studies of integrin clustering is that in the fly, this process appears to occur independently of activation. In comparison, a number of mammalian integrin studies have found a mechanistic interdependence between clustering and activation [44]. This is because both processes were hypothesized to depend on separation of the salt bridge that forms between the α and β-integrin subunits [49]. However, only a subset of mutations that disrupt integrin activation also abrogate integrin clustering [21]. Based on contradictory results from studies in keratinocytes and platelets, we speculate that the relationship between activation and clustering might depend on the type of ECM ligand involved. It is known that blood cells such as platelets encounter soluble ligands and depend on talin-dependent control of integrin affinity as well as integrin clustering, to stably bind ligand and initiate a clotting response [3], [4]. In contrast, keratinocytes in skin face insoluble, high-density ligands [50]–[52] and mutations in β-integrin that strongly disrupt integrin activation and ligand binding have no effect on integrin function [6]. Importantly, in keratinocytes, there is a known requirement for integrin clustering [5], [7]. We propose that, in the context of fly development, integrins typically encounter a high density of insoluble ECM ligands and thus primarily regulate their ligand-binding function by receptor clustering rather than through activation.

The *rhea^17^* mutant allele affects clustering as well as reinforcement and maturation of cell-ECM adhesions. This introduces the possibility that these two events are linked. Such linkage might occur because integrin clustering can create a concentrated platform for adhesion complex formation and maintenance. Subsequently, it is reasonable to postulate that integrin clustering promotes robust adhesion complex assembly and maturation. In line with this prediction, in *rhea^17^* mutant embryos, we observed progressive loss of adhesion complex components from MTJs between during an important developmental transition (stages 16–17). During this period of growth, sarcomeres form and muscle contraction begins and in wild-type embryos the recruitment of talin and other adhesion complex components increases dramatically to provide resistance to the growing tensile forces ([37], this study). We anticipate that as increased force is exerted on integrin-mediated adhesions at MTJs, the talin head is required to facilitate adhesion reinforcement. If the stability of the talin head at adhesions is compromised, as in *rhea^17^*, this reinforcement cannot occur leading to the disintegration of MTJs and subsequent muscle detachment in stage 17, which is precisely what we observed. In further support of this hypothesis, we discovered that MTJs were longer in *rhea^17^* mutant embryos, suggesting that in the absence of effective integrin clustering, adhesions are not able to consolidate into tight, compact MTJs able to support the forces of muscle contraction.

Both in *Drosophila* and in mammalian cell culture, mutations have been identified that prevent integrin binding but do not prevent integrin clustering [21], [25]. This observation raises the question of whether the talin head must bind to integrin in order to induce clustering and adhesion reinforcement. One possibility is that the talin head binds to integrins via its C-terminal IBS-2 domain, freeing up the head to act as a scaffold for other adhesion complex components. In many ways, the phenotype caused by the *rhea^17^* mutation resembles the effect of disrupting of the IBS-2 domain [24]; in both cases, attachment between integrins and the actin cytoskeleton is severely compromised. Perhaps F2-F3 interactions are required not only for coordinated integrin and membrane binding, but also for talin head binding to other adhesion complex proteins. This hypothesis constitutes an intriguing avenue of future study.

In summary, our results provide insights into how integrins are regulated under physiological conditions to give rise to stable tissue architecture. Our work suggests that the canonical model of talin head function as an integrin activator should be modified to include an additional essential role as an orchestrator of integrin clustering and adhesion complex reinforcement. We furthermore illustrate how specific inter-domain interactions in the talin head contribute to the regulation of integrin function. Based on our data we propose the following model for talin function in the fly embryo: talin is recruited to integrin initially through its IBS2 domain (see [24]), which helps assemble an adhesion complex that links to the cytoskeleton. During embryogenesis, there is an increasing need to generate stronger adhesion as the growth of the embryo generates proportionally greater mechanical strain upon the tissues. It is at this point that clustering becomes essential. Talin is then recruited to integrins via its head domain and is stabilized within growing adhesive contacts by coordinated interactions between F2-F3 and the plasma membrane. These stabilized talin-head-integrin complexes form clusters and act as a scaffold for adhesion complex assembly and cytoskeletal attachment that is maintained and reinforced throughout tissue growth and development. Failure to cluster integrins results in severe defects in reinforcement of cell adhesion, and subsequently Cell-ECM adhesions breakdown in the face of increasing mechanical force [31]. Thus, our work sheds novel light on the molecular mechanisms that act through talin to promote adhesion receptor clustering and adhesion complex stability, crucial aspects underlying tissue morphogenesis and homeostasis.

## Materials and Methods

### Molecular biology

The generation of talinGFP is previously described [30]. To make pUbi-talinEGFP*L334R mutant construct, pBS-talinGFP was mutated using the QuikChange Lightning mutagenesis kit (Stratagene). The talinGFP*L334R cassette was sub-cloned into the pUbi63E vector using a strategy similar to that used to generate the WT talinGFP construct [30]. The making of pUASp-GFP-TalinHead was described previously [25]. This construct was directly mutated to contain the L334R point mutation using the QuikChange mutagenesis kit (Stratagene).

### Fly stocks and genetics

All rescue experiments were performed in mutant background such that both maternal and zygotic contributions of talin were eliminated, using the *rhea^79^* allele and the Dominant Female Sterile technique [29]. The *rhea^79^* allele was generated by a P-element excision that covers the entire rhea locus. See [32] for a complete characterization. Females of the genotype yw, hs-Flp/+; pUbi-talinGFP, talinGFP*L334R, or headless-TalinGFP/+; rhea79a, FRT2A/OvoD1, FRT2A were subjected to a heatshock-regime during the larval stages to generate a mosaic germline in order to give rise to *rhea* mutant oocytes with maternally supplied rescued transgenes. Virgins were then crossed to *rhea^79a^/TM6b, dfd-GMR-nvYFP* males. Embryos without the fluorescent balancer were selected for analyses. Using this approach we find that WT talinGFP rescued embryos resemble WT embryos and that over-expression of transgenic talin does not cause any deleterious effects or ectopic signaling integrin ([24], [25]; Supplemental Fig. S5).

The *rhea^17^* allele was sequenced according to conventional protocols by sequencing of the entire rhea coding sequence (see Supplemental Table S1 for a list of primer pairs). The G340E mutation was identified in exon 5 through comparison of genomic DNA from homozygous wild type OR flies and heterozygous *rhea^17^* flies (Supplemental Fig. S3). Maternal-zygotic *rhea^17^* mutants were generated via the Dominant Female Sterile germline-clone technique and crossed to *rhea^17^/TM3, dfd-GMR-nvYFP* males or, to assess the phenotype of *rhea^17^* over a deficiency, to *rhea^79^/TM3, dfd-GMR-nvYFP* males.

For all talin FRAP experiments, talinGFP constructs were heterozygous and expressed in a *w^1118^* background. For integrin-YFP FRAP experiments, the transgene was either expressed in a heterozygous *w^1118^* background (neomycin experiments), or expressed in a *rhea* mutant background (either *rhea^17^* or *rhea^79^*).

*UAS*-driven transgenes were expressed in the muscle using the muscle-specific *mef2*-Gal4 driver.

For analysis of integrin clustering with talin transgenes, *yw, hsFLP;;GFP-FRT2A* virgins were crossed to males of the genotype of either *rhea79, FRT2A/TM3, dfd-GMR-nvYFP*, *headlessTalin-GFP/Y;;rhea79/TM3, dfd-GMR-nvYFP, L334R; rhea79 FRT2A/TM3, dfd-GMR-nvYFP* or *rhea17, FRT2A/TM3, dfd-GMR-nvYFP*. For analysis of integrin clustering with integrin transgenes *ubi-GFP,FRT101; hsFLP* males were crossed to virgin progeny from *mys^XG43^, FRT101/FM7, Kr>GFP* crossed to males of the genotype Ubi-integrinYFP*D807R or UBi-integrinYFP*G792N. For both integrin and talin transgenes larval progeny were subject to a heat-shock regime in order to induce clones. Wandering third instar larvae were selected for dissection and analysis.

### Confocal immunofluorescence imaging and image analysis

Embryos and third instar imaginal wing discs were fixed and stained according to standard protocols. The following antibodies were used in our analysis: rabbit anti-talin (1∶500), mouse anti talin (1∶50; DSHB) mouse monoclonal anti- βPS-integrin (1∶50; DSHB), rat anti-αPS2-integrin (1∶200, 7A10), mouse anti-tiggrin (1∶1000; Liselotte Fessler, UCLA), mouse anti-Myosin Heavy Chain (1∶200; Dan Kiehart, Duke University), rabbit anti-PINCH (1∶1000; Mary Beckerle, University of Utah), rabbit anti-phospho-FAK (1∶200; Invitrogen) and rabbit anti-paxillin (1∶1000; [53]). Rhodamine-conjugated phalloidin (Invitrogen) was used to stain actin filaments (1∶400). Fluorescently- conjugated Alexa-Fluor-488, Cy3 and Cy5 secondary antibodies were used at 1∶400 dilution (Molecular Probes). Images were collected using an Olympus FV1000 inverted confocal microscope and an UplanFL N 40×1.30 NA oil objective or a UplanSApo 60×1.35 NA objective. For all micrographs of whole embryos, or of MTJs, z-stacks were assembled from 8–12 1.0–2.0 µm confocal sections. Embryos were staged as described in [23]. For the line scan analyses in Fig. 1, fluorescence intensity profiles across the boxed areas were obtained using ImageJ (NIH, Bethesda, MD) and then normalized to the maximum fluorescence intensity in each channel. See also [24]. Recruitment of integrin, talin, or talinGFP to MTJs was calculated according to our previously established method ([23]–[26], [35], [36]). Briefly, the mean fluorescence intensity of the signal of interest was measured at the MTJ and the cytoplasm and a ratio of MTJ∶cytoplasmic signal was determined. This value was averaged based on measurements of 5 MTJs (all ventral-lateral attachments from hemi-segments A2–A6) from at least 3 embryos. Localization of IAC components to MTJs was quantified as follows, adapted from the method described in [37]: for each IAC component (talin, PINCH, and pFAK), recruitment was determined by measuring the mean fluorescence intensity at MTJs, which was then expressed as a ratio over the mean fluorescence of **α**PS2-integrin staining at each MTJ. For each genotype, at 5 MTJs were measured from at least 3 embryos. MTJ length was assessed by measuring the length∶width ratio of the D-V length of ventral-lateral MTJ over the A-P width of adjacent VL1 muscles in the hemisegment posterior to each MTJ that was measured. All images were taken using the same gain and offset settings. This value was averaged based on measurements of 5 MTJs (hemi-segments A2–A6) from at least 3 embryos. All quantitative analyses were obtained using ImageJ (NIH, Bethesda, MD) and two-sided Student's t-tests were performed using Prism5 (GraphPad Software Inc., La Jolla, CA).

### FRAP

Stage 17 embryos were collected and prepared for FRAP as described previously [30]. Briefly, embryos were collected from apple juice plates, dechorinated in 50% bleach for 4 minutes, washed with PBS and mounted onto glass slides in PBS. FRAP analysis was performed at room temperature. Photo-bleaching was performed using a 473 nm laser at 5% power with the Tornado scanning tool (Olympus) for 2 seconds at 100 mseconds per pixel. Fluorescence recovery was recorded over 5 minutes at 1 frame every 4 seconds. To control muscle twitching in and out of focus, multiple regions of interest (ROIs) were selected in non-photobleached regions; only samples for which intensities within control ROIs remained steady throughout the FRAP experiment were used. The mobile fraction and statistical tests were performed using Prism 5 software. Neomycin treatment, which was used to inhibit integrin clustering through sequestration of PI(4,5)P2 phospholids [19], [38], [39], was carried out according to the embryonic drug delivery protocol described in [54], in *w^1118^* embryos expressing *ubi*-integrinYFP [30]. The control for integrin FRAP in *rhea^17^* zygotic mutant embryos was *rhea79/+* embryos expressing *ubi*-integrinYFP. We have previously observed that genetic background influences the baseline turnover of integrin adhesion complex components and therefore each experiment requires its own control [31]. In particular, we find that the drug delivery protocol lowers turnover in vehicle-only treated embryos, and thus explains the differences in the mobile fractions of the controls shown in Fig. 4k versus Fig. 4l.

### Western blots

Westerns were carried out as previously described [17]. Animals assayed either were heterozygous for the *rhea^79^* talin null allele or the *rhea^17^* allele. Antibodies used were rabbit anti-talin antibody (1∶2000; [26]) and mouse monoclonal anti-β-actin (1∶5000, AbCam 8224).

### Protein expression and purification

All constructs were expressed in E. coli BL21 Star (DE3) cultured in 2YT media. GST-tagged talin head recombinant proteins (residues 1–409) and mutants therein were purified using glutathione sepharose resin (GE Healthcare) and eluted by TEV cleavage. Protein concentrations were determined using extinction coefficients at 280 nm.

### Mass spectrometry

Proteomics were carried out by the University of Leicester Proteomics Facility (PNACL, University of Leicester) essentially as described previously [55]. The TEV eluted proteins were run on an SDS-PAGE gel and the corresponding bands were excised. The gel stab was digested with trypsin and subjected to LC-MS/MS mass spectrometry using an RSLCnano HPLC system (Dionex, UK) and an LTQ-Orbitrap Velos mass spectrometer (Thermo Scientific). The data were analysed using Mascot (Matrix Science Ltd.) and Scaffold (Proteome Software). The identified peptide fragments were visualized on a structural model of fly talin head produced using the mouse talin1 head construct.

### Structural modeling

All structure images were generated with PyMol (PyMOL Molecular Graphics System, Version 1.5.0.4 Schrödinger, LLC.). The modeling of the potential consequence of the G340E mutation was carried out using the atomic structure of the mouse talin2 F2F3 domain in complex with the beta1d cytoplasmic tail (PDB: 3G9W [15]). The residues that define the extensive interface between the F2 and F3 interface are conserved between mouse and fly with G340 crucial to enable close packing of the two domains. Visualization of the G340E mutant was made using the mutagenesis function in PyMol.

### Analysis of αIIbβ3 Integrin Activation

The activation state of αIIbβ3 integrins was assessed by measuring the binding of the ligand mimetic anti-αIIbβ3 monoclonal antibody PAC1 in flow cytometric assays as described previously [56]. A CHO cell line stably expressing αIIbβ3 [57], [58] was transfected with the indicated GFP tagged fly talin head cDNA using polyethylenimine (PEI) and 18 h later cells were suspended and stained with αIIbβ3 integrin activation-specific PAC1 IgM (BD Biosciences) in the presence and absence of the ligand binding inhibitor EDTA (Sigma). αIIbβ3 integrin expression was assessed separately by staining with monoclonal antibody D57 [57], a gift from M. Ginsberg (UCSD). Cells were washed and PAC1 binding to live, transfected (GFP-positive) cells was assessed with Alexa647-conjugated goat anti-mouse IgM (Invitrogen). In parallel, bound D57 to live expressing cells with similar GFP fluorescence intensity was detected using Alexa 647 fluorophore-conjugated goat anti-mouse IgG (Invitrogen). Activation was quantified and an activation index was calculated as defined by the formula AI = (F – F0)/(Fintg), where F is the geometric mean fluorescence intensity (MFI) of PAC1 binding, F0 is the MFI of PAC1 binding in the presence of EDTA, and Fintg is the standardized ratio of D57 binding to transfected cells. The Fintg expression ratio was defined as follows: Fintg = (Ftrans)/(Funtrans), where Ftrans is the geometric MFI of D57 binding to GFP-positive cells and Funtrans is the MFI of D57 binding to untransfected cells. FACS data analysis was carried out using FlowJo FACS analysis software and statistical analysis using GraphPad Prism software.

## Supporting Information

Figure S1 Muscle detachment phenotypes in IBS-1 mutant embryos. (**a**) Phenotypic scoring of talin*R367A-rescued embryos revealed about 75% of animals displayed muscle attachments defects. (**b**) Late, mild muscle detachment was observed in stage 17 R367A-rescued embryos stained for MHC to mark the muscle cytoskeleton and talin. Compare to the milder phenotype of the talin*L334R rescued embryo shown in Fig. 1.
(PDF)

Figure S2 Quantification of protein levels of transgenic talin rescue constructs. Western blotting analysis was used to quantify protein levels of each transgene used in this study. Previous study revealed that expression of WT talinGFP was expressed at levels nearly twice as high as endogenous talin, and that expression at this level was sufficient to rescue all of the phenotypes associated with loss of talin (see Ellis et al, 2013). Although the headlessTalinGFP expression levels appeared lower, no significant differences in expression level were found between WT talinGFP and talinGFP*L334R (p = 0.5193), or between WT talinGFP and headlessTalinGFP. (p = 0.2682).
(PDF)

Figure S3 Identification of the genetic lesion responsible for the *rhea^17^* allele. Comparison of multiple sequence reads over exon 5 of the rhea locus for wild type flies and rhea17 heterozygous flies uncovered potential single nucleotide polymorphisms (SNPs) in WT and *rhea^17^* alleles of talin. The first SNP shown was found to be a silent mutation that did not result in a change to the coding sequence. A second SNP caused a g>a base pair substitution resulting in a missense mutation (G340E) in the coding sequence of the *rhea^17^* allele. This base pair substitution was observed over multiple reads.
(PDF)

Figure S4 Mutations that impinge on conformational changes to the transmembrane and intracellular domains of β-integrin do not affect integrin clustering in *Drosophila*. (a) Mosaic analysis of integrin mutant clones in third instar larval wing imaginal discs dissected from embryos in which βPS−integrinYFP transgenes containing either the D807R (a) or G792N (b) point mutations were ubiquitously expressed. Both mutations rescued the formation of basally localized integrin clusters (a′, b′) in integrin mutant clones (marked by loss of GFP in a, b). (a″–b″) Quantification of the density of basal integrin clusters failed to reveal any significant differences between the density of clusters in control tissue versus mutant tissue expressing either β-integrinYFP*D807R (a″) or β-integrinYFP*G792N (b″). Scale bar = 10 µm.
(PDF)

Figure S5 Effects of talin overexpression on muscle attachment. (a,b) Whole mount stage 17 embryos stained with phalloidin to label F-actin demonstrate that over-expression of talin using the muscle specific Mef2-Gal4 driver does not significantly affect muscle morphology. No embryos in either control mef2-GAL4 (a; n = 78) or mef2>UAS-talin muscles (b; n = 43) showed muscle attachment defects. Scale bars = 50 µm.
(PDF)

Table S1 Primer pairs used for sequencing of the *rhea* locus. The g>a mutation underlying the G340E mutation in the *rhea^17^* mutant allele was uncovered using primer pair 5.
(XLSX)
