# Supplementary figures and images for: The Talin Head Domain Reinforces Integrin-Mediated Adhesion by Promoting Adhesion Complex Stability and Clustering
Source: PLoS Genet. 2014 Nov 13;10(11):e1004756. doi: 10.1371/journal.pgen.1004756 (PMC4230843; doi:10.1371/journal.pgen.1004756)

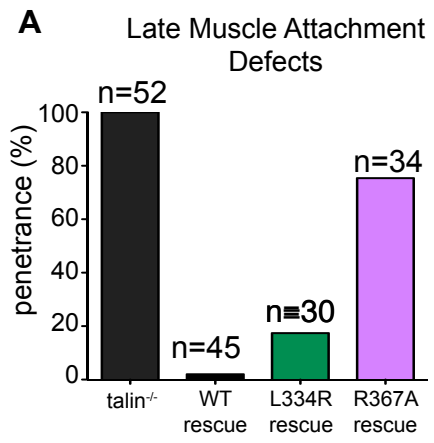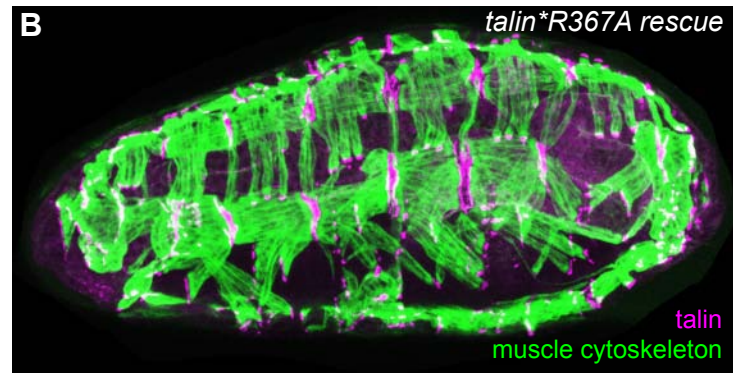

Supplement: Figure S1 — Muscle detachment phenotypes in IBS-1 mutant embryos. (a) Phenotypic scoring of talin*R367A-rescued embryos revealed about 75% of animals displayed muscle attachments defects. (b) Late, mild muscle detachment was observed in stage 17 R367A-rescued embryos stained for MHC to mark the muscle cytoskeleton and talin. Compare to the milder phenotype of the talin*L334R rescued embryo shown in Fig. 1. (PDF) [file pgen.1004756.s001.pdf]

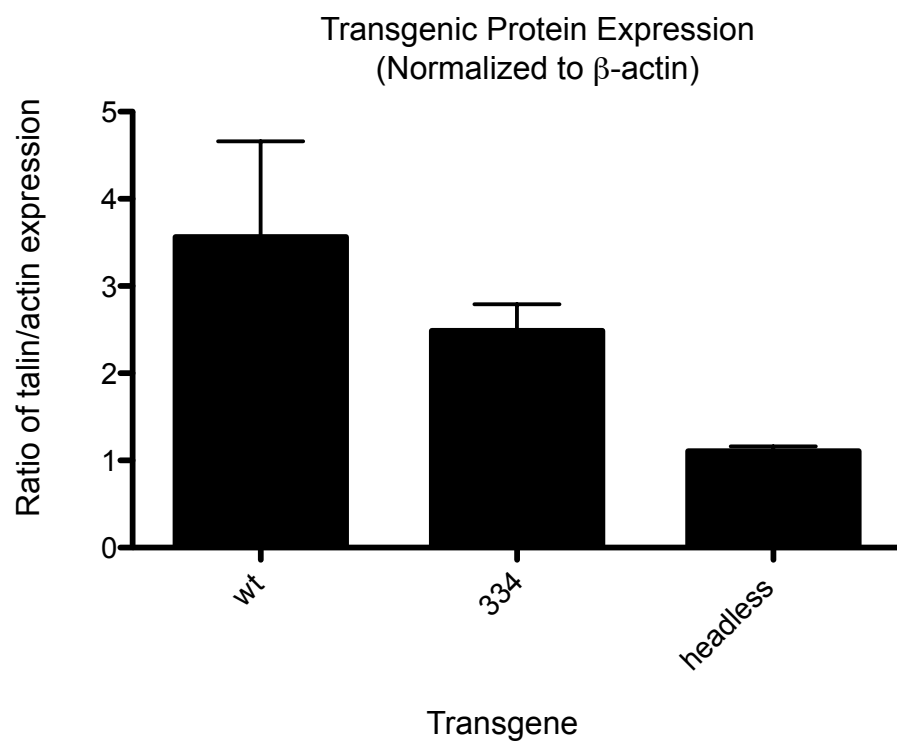

Supplement: Figure S2 — Quantification of protein levels of transgenic talin rescue constructs. Western blotting analysis was used to quantify protein levels of each transgene used in this study. Previous study revealed that expression of WT talinGFP was expressed at levels nearly twice as high as endogenous talin, and that expression at this level was sufficient to rescue all of the phenotypes associated with loss of talin (see Ellis et al, 2013). Although the headlessTalinGFP expression levels appeared lower, no significant differences in expression level were found between WT talinGFP and talinGFP*L334R (p = 0.5193), or between WT talinGFP and headlessTalinGFP. (p = 0.2682). (PDF) [file pgen.1004756.s002.pdf]

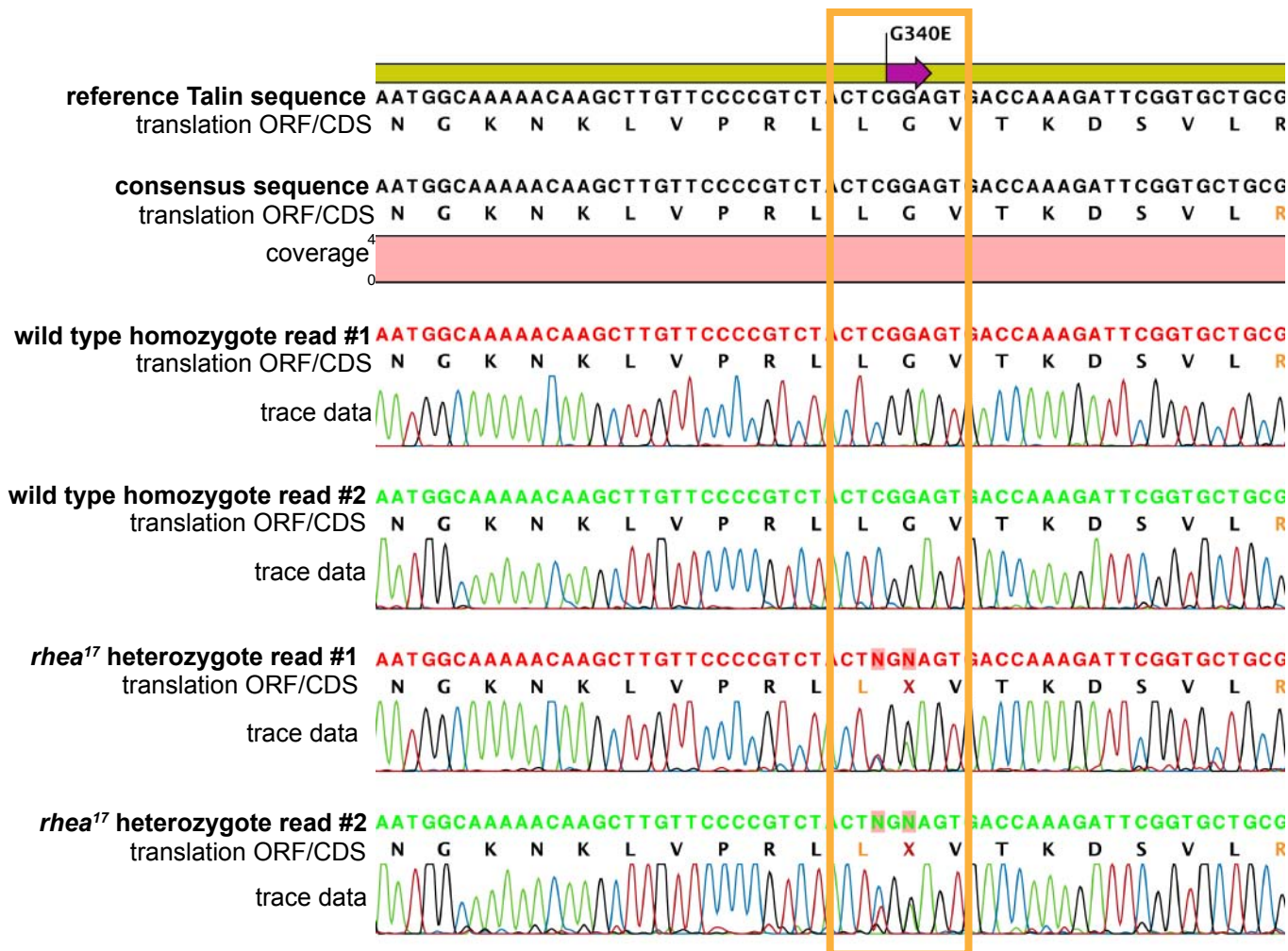

Supplement: Figure S3 — Identification of the genetic lesion responsible for the rhea17 allele. Comparison of multiple sequence reads over exon 5 of the rhea locus for wild type flies and rhea17 heterozygous flies uncovered potential single nucleotide polymorphisms (SNPs) in WT and rhea17 alleles of talin. The first SNP shown was found to be a silent mutation that did not result in a change to the coding sequence. A second SNP caused a g>a base pair substitution resulting in a missense mutation (G340E) in the coding sequence of the rhea17 allele. This base pair substitution was observed over multiple reads. (PDF) [file pgen.1004756.s003.pdf]

*mys*<sup>XG43</sup> + *pUbi-integrin YFP\*D807R*

*mys*<sup>XG43</sup> + *pUbi-integrin YFP\*G792N*

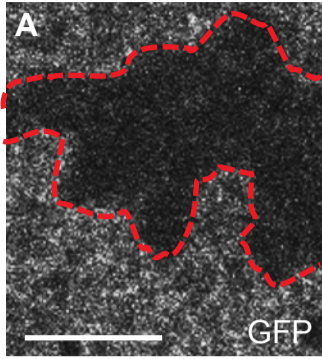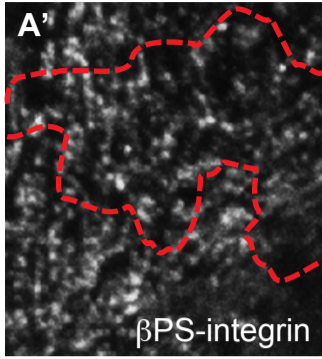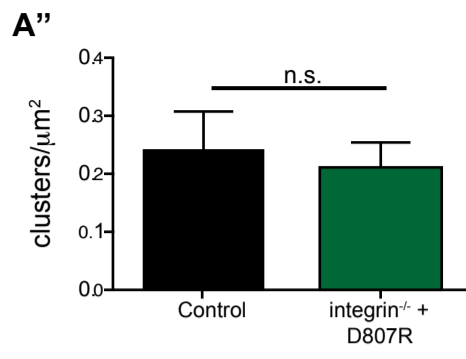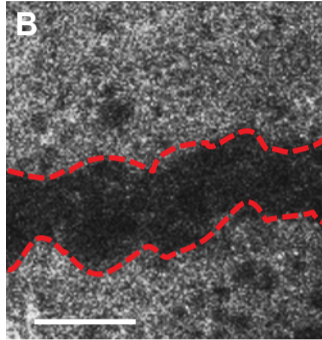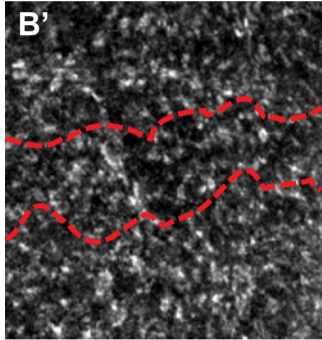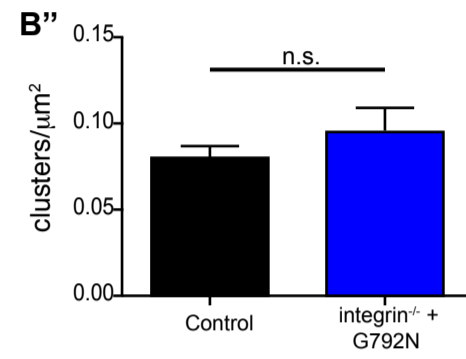

Supplement: Figure S4 — Mutations that impinge on conformational changes to the transmembrane and intracellular domains of β-integrin do not affect integrin clustering in Drosophila. (a) Mosaic analysis of integrin mutant clones in third instar larval wing imaginal discs dissected from embryos in which βPS−integrinYFP transgenes containing either the D807R (a) or G792N (b) point mutations were ubiquitously expressed. Both mutations rescued the formation of basally localized integrin clusters (a′, b′) in integrin mutant clones (marked by loss of GFP in a, b). (a″–b″) Quantification of the density of basal integrin clusters failed to reveal any significant differences between the density of clusters in control tissue versus mutant tissue expressing either β-integrinYFP*D807R (a″) or β-integrinYFP*G792N (b″). Scale bar = 10 µm. (PDF) [file pgen.1004756.s004.pdf]

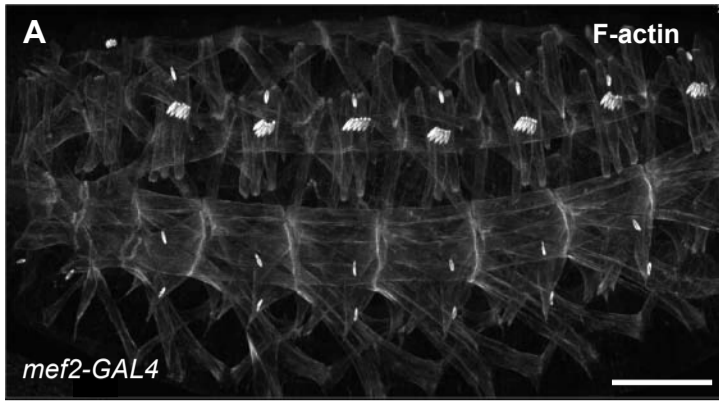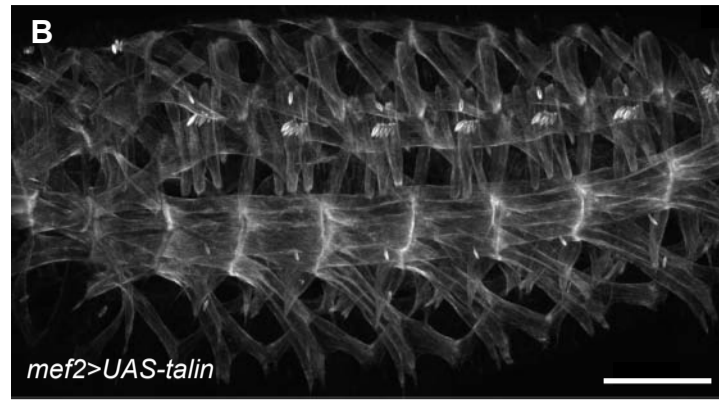

Supplement: Figure S5 — Effects of talin overexpression on muscle attachment. (a,b) Whole mount stage 17 embryos stained with phalloidin to label F-actin demonstrate that over-expression of talin using the muscle specific Mef2-Gal4 driver does not significantly affect muscle morphology. No embryos in either control mef2-GAL4 (a; n = 78) or mef2>UAS-talin muscles (b; n = 43) showed muscle attachment defects. Scale bars = 50 µm. (PDF) [file pgen.1004756.s005.pdf]
